# Molecular mechanisms governing microRNA-125a expression in human hepatocellular carcinoma cells

**DOI:** 10.1038/s41598-017-11418-3

**Published:** 2017-09-06

**Authors:** Nicoletta Potenza, Marta Panella, Filomena Castiello, Nicola Mosca, Elena Amendola, Aniello Russo

**Affiliations:** 1 Department of Environmental, Biological and Pharmaceutical Sciences and Technologies, University of Campania Luigi Vanvitelli, Via Vivaldi 43, 81100 Caserta, Italy; 2 Department of Molecular Medicine and Medical Biotechnology, University of Naples Federico II, Via Pansini 5, 80131 Napoli, Italy

## Abstract

MicroRNA-125a-5p (miR-125a) is a vertebrate homolog of lin-4, the first discovered microRNA, and plays a fundamental role in embryo development by downregulating Lin-28 protein. MiR-125a is also expressed in differentiated cells where it generally acts as an antiproliferative factor by targeting membrane receptors or intracellular transductors of mitogenic signals. MiR-125a expression is downregulated in several tumors, including hepatocellular carcinoma (HCC) where it targets sirtuin-7, matrix metalloproteinase-11, VEGF-A, Zbtb7a, and c-Raf. In this study, we have isolated the transcription promoter of human miR-125a and characterized its activity in HCC cells. It is a TATA-less Pol II promoter provided with an initiator element and a downstream promoter element, located 3939 bp upstream the genomic sequence of the miRNA. The activity of the promoter is increased by the transcription factor NF-kB, a master regulator of inflammatory response, and miR-125a itself was found to strengthen this activation through inhibition of TNFAIP3, a negative regulator of NF-kB. This finding contributes to explain the increased levels of miR-125a observed in the liver of patients with chronic hepatitis B.

## Introduction

MicroRNAs (miRNAs) are small non-coding RNAs that play crucial roles in regulating gene expression in a variety of physiological processes by affecting both translation and stability of complementary mRNAs^1, 2^. Over the past decade, several studies have been devoted to quantitative and qualitative assessment of miRNA expression, showing that miRNA abundance is tightly regulated during development and across tissues. These results have also shown that aberrant expression of miRNAs is linked to pathological conditions, pointing to the miRNA profiling as an important tool for diagnostics and treatment of diseases.

The most remarkable changes in miRNA expression are observed in cancer^3, 4^. Lowered expression of the microRNA biosynthesis enzyme Dicer in tumor cells or mutations in its structure often lead to altered biosynthesis of microRNAs and increased tumorigenesis^5–8^. On the other hand, Dicer expression often increases during cell differentiation^9–11^. In this field, a growing body of evidence indicates that dysregulated expression of specific miRNAs plays a causative role, since miRNAs can function as either tumor suppressors by down-regulating oncogenic targets, or tumor promoters by negatively regulating oncosuppressor proteins^12–14^. However, less attention has been paid to the molecular mechanisms regulating miRNA expression.

MicroRNAs are transcribed by RNA polymerase II as long primary transcripts (pri-miRNAs) that are 5′-capped and 3′-polyadenylated^15^. Pri-miRNAs may extend hundreds of kilobases in length and are either monocistronic, i.e. one miRNA for transcription unit, or polycistronic, i.e. a cluster of miRNAs for transcription unit. Genomic miRNA sequences may be hosted by coding or non-coding genes, generally sharing their transcription promoter. Either way, the mature miRNA sequences are located within regions that fold into hairpin structures, recognized and excised by Drosha and DGCR8, the microprocessor complex, generating 60–80 nt precursors (pre-miRNAs). Pre-miRNAs are exported to the cytoplasm where they are processed by Dicer in miRNA duplexes. Finally, the mature miRNA strand is loaded onto an Argonaute protein within the RISC complex to bind and silence complementary mRNA targets.

MircroRNA-125a-5p (miR-125a), denominated lin-4 in nematodes, is of special interest, since it is very well conserved in evolution^16^ and plays a pivotal role in development and cell differentiation^1, 17–20^. Expression of this miRNA generally increases with cell differentiation whereas it is downregulated in several types of tumors, including breast^21–23^, gastric^24^, cervical^25^, lung^26, 27^, ovarian^28^, and colon^29^ cancers, retinoblastoma^30^, medulloblastoma^31^, glioblastoma^32^, neuroblastoma^33^, and hepatocellular carcinoma^34–36^.

Hepatocellular carcinoma (HCC) is the third cause of cancer-related deaths and the fifth most common cancer worldwide^37, 38^. Few miRNAs have been shown to play an oncosuppressive role in HCC^39, 40^. Among them, miR-125a inhibits cell proliferation, angiogenesis and cell migration by downregulating the expression of sirtuin-7^34^, vascular endothelial growth factor A, matrix metalloproteinase-11^35^, Zbtb7a^41^, and c-RAF^42^. Targeting of Bcl2 and caspase 3 may also be relevant for the antiangiogenic activity^43^ of the miRNA. Experimental up-regulation of miR-125a by lentivirus-mediated transfection of HCC cells limited cell proliferation and tumor growth in nude mice. Moreover, low tumor expression of miR-125a at time of surgery in HCC patients has been correlated with poor 5-year survival^35^. Although the role of miR-125a during the carcinogenesis is object of extensive research, the mechanisms governing miR-125a expression are still largely unexplored.

In this study we identified and functionally characterized the promoter of the transcription unit of miR-125a, linking its activity to the transcription factor NF-kB and the inflammatory response.

## Results and Discussion

### Identification of miR-125a promoter

**Figure 1:**
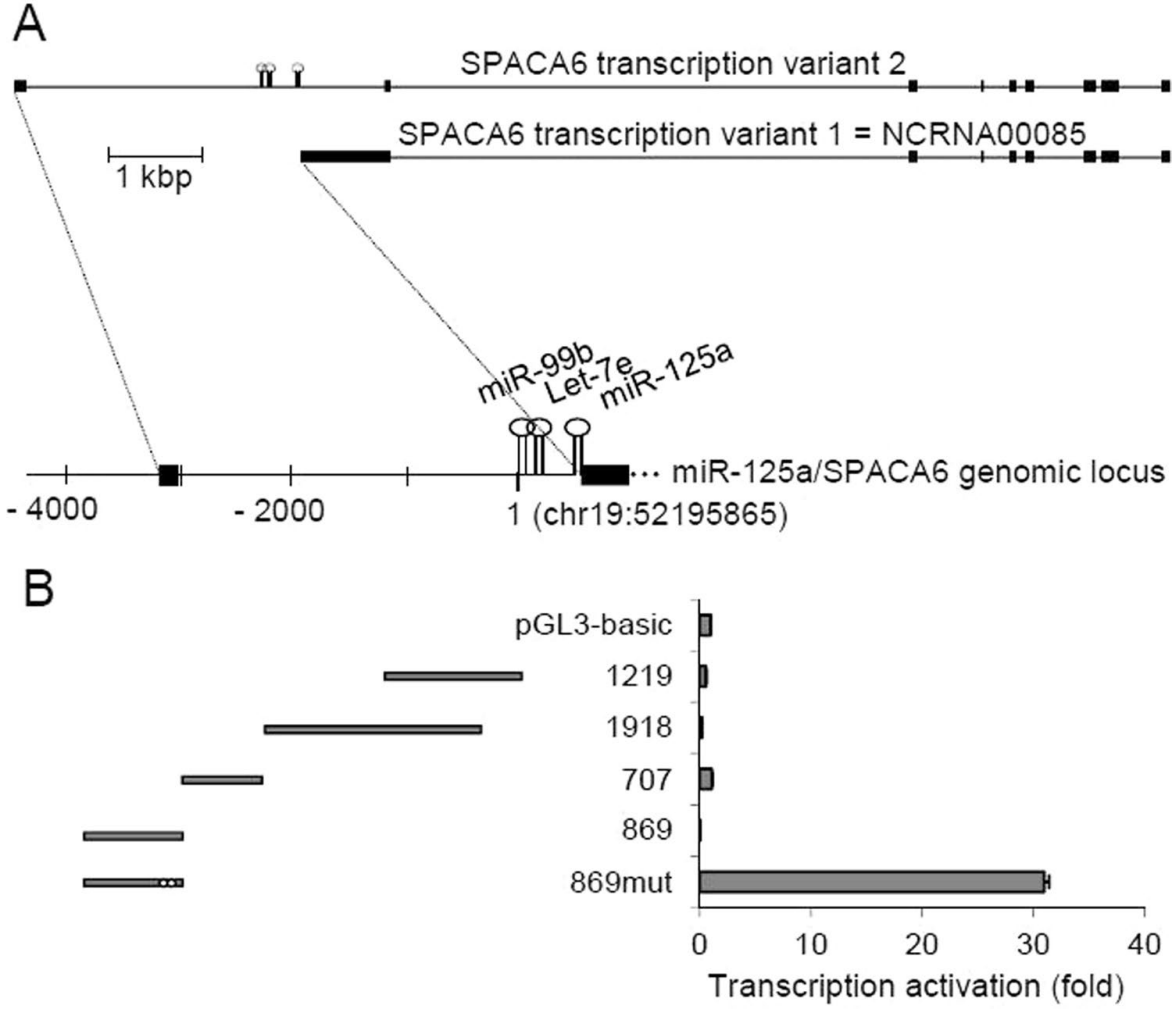
Isolation of miR-125a promoter. (**A**) Two major transcripts of SPACA6 gene and map of its genomic locus; exons and microRNAs are indicated by black boxes and loops, respectively; the first base of pre-miR-99b was assigned as nucleotide 1 (**B**) Five genomic DNA segments (grey bars in the left side of the panel) spanning nucleotides -36 to -3875 were cloned in the luciferase reporter plasmid pGL3-basic and assayed for transcription promoter activity in HepG2 cells. The reporter constructs are named according to the size of the cloned genomic fragment and their activity is reported in the adjacent plot; 869mut construct carries two point mutations (marked by white dots) eliminating putative translation start sites. Assays were performed at least in triplicate and expressed as mean ± SD.

**Figure 2:**
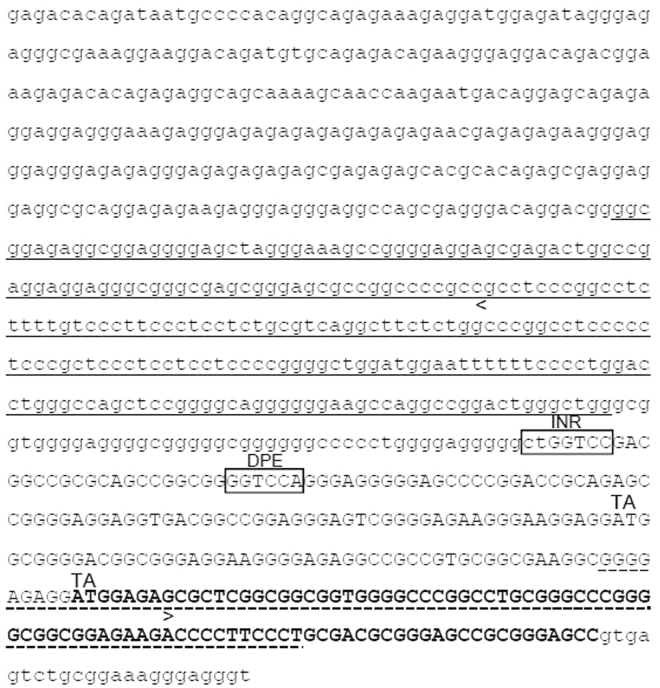
Nucleotide sequence of miR-125a/SPACA6 promoter. The sequence encompasses the 869 bp genomic segment located 3006 nucleotides upstream of pre-miR-99b that showed transcription promoter activity in the luciferase assay. INR, initiator element; DPE, downstream promoter element; uppercase, exonic sequence; bold, coding sequence; underlined, proximal enhancer containing putative binding sites for Sp1, AP-2, T-Ag, SIF, GCF, and ATF (PROSCAN 1.7); dashed underlined sequence, NF-kB binding region; TA, point mutations yielding 869mut construct. The nucleotide sequence between <and> shows an high degree of identity (82%) with the corresponding mouse genomic sequence. Nucleotides 371–381 contain methylation sites affecting miR-125a expression in acute myeloid leukemic cells, as shown by the de-methylating agent decitabine, whose cell treatment increased miR-125a expression by more than 10-fold^65^.

The genomic sequence of miR-125a-5p is located on chromosome 19, in close proximity to those of let-7e and miR-99b (Fig. 1A). This cluster was originally reported to be placed immediately upstream of the first annotated exon of NCRNA00085, a long non-coding RNA with unknown function. Later, based on sequence homology with mouse genome, an ORF was found and it became evident that the transcript encodes a polypeptide chain of 324 amino acid residues, denominated sperm acrosome-associated 6 (SPACA6) protein, that in mice is implicated in sperm-egg fusion during fertilization^44^. More recently, a new exon of SPACA6 gene has been annotated^45^. It is located 3029 bp upstream of human pre-miR-99b and belongs to a transcript encoding a different isoform of SPACA6 protein of 283 amino acid residues, denominated isoform 2 (Fig. 1A). Based on these data, it may be hypothesized that the miR-125a cluster 1) is provided with its own promoter, located within the first intron of SPACA6 gene, or 2) shares with SPACA6 a promoter located upstream of the first exon. To verify these hypotheses, different genomic segments, spanning nucleotides -36 to -3875 (with the first nucleotide of pre-miR-99b assigned as 1), were isolated by PCR from genomic DNA and cloned upstream the coding sequence of firefly luciferase into pGL3-basic promoterless vector (Fig. 1B). Surprisingly, none of the constructs yielded significant luciferase activity after transfection in HepG2 cells. Inspection of the same genomic region by PromoterScan predicted a top-ranking promoter upstream of the first exon, within the 869 segment (Fig. 1B). Also the program miRStart identified putative transcription start sites (TSS) in the same segment. Further analysis of the same sequence revealed that following the TSSs there were two translation start codons within Kozak sequences (both 5′-ACCATGG-3′) separated by 59 bp. We then considered the possibility that the 869 segment may contain both the SPACA6 transcription promoter and the translation start site; its cloning in the luciferase reporter plasmid would have then directed the transcription of a chimeric mRNA whose translation couldn’t be initiated at the luciferase start site but 100–150 bp upstream, leading to translation frameshift and/or production of an inactive fusion protein. To test this hypothesis, the two putative translation start sites were removed by site-directed mutagenesis and the resulting DNA segment, 869mut, was assayed for promoter activity in HepG2 cells. This assay revealed a strong luciferase activity with a 31-fold activation of the reporter gene, compared to the parental vector pGL3-basic (Fig. 1B). When assayed in HuH7 hepatocarcinoma cells, promoter activity was increased to 43-fold. These data strongly suggest that the genomic DNA sequence located between -3875 and -3006 bp from pre-miR-99b drives the expression of SPACA6 gene and its intronic miR-99b/let-7e/miR-125a cluster. The nucleotide sequence of the 869 segment with annotation of the putative promoter elements is reported in Fig. 2.

### Characterization of miR-125a/SPACA6 promoter

**Figure 3:**
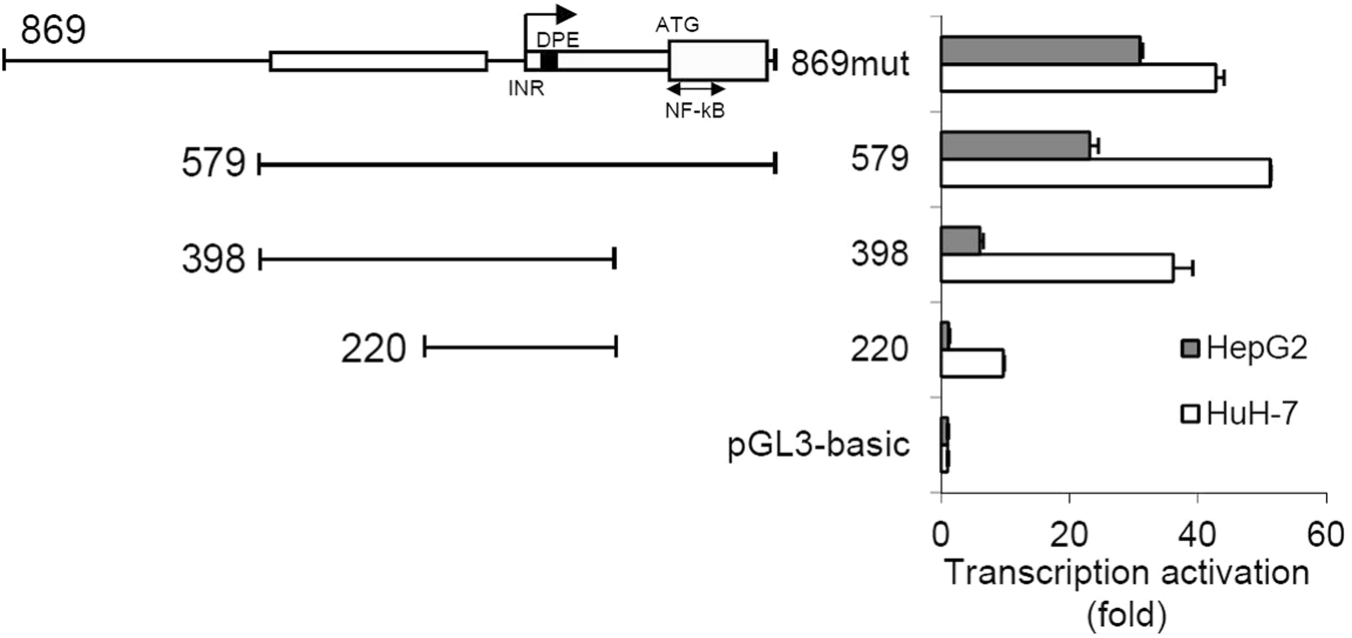
Refinement of the promoter map by deletions. Deletion mutants of 869mut were transfected in HepG2 and HuH-7 cells and assayed for promoter activity. Specific sections of 869mut are marked as follows: INR, initiator element; DPE, downstream promoter element; NF-kB, NF-kB binding region; open box, proximal enhancer, grey boxes, exonic sequences; thick box, coding sequence. Assays were performed in triplicate and expressed as mean ± SD.

In order to determine the minimal sequence required for transcription, a series of deletion mutants of 869mut were generated and assayed for promoter activity in HepG2 and in HuH-7 cells. Deletion of 290 bp from the 5′-end of 869mut, generating the 579 construct, had no effect on promoter activity, consistent with the extension of the predicted proximal enhancer (Fig. 3). Further deletion of 181 bp from the 3′-end of 579 construct, generating the 398 construct, reduced the luciferase activity by 70% in HepG2 and 30% in HuH-7 cells, implying that the deleted sequence contains regulatory elements that enhance transcription. An additional deletion of the 5′-end of 398 segment, yielding the 220 construct, drastically affected the activation of the reporter gene, presumably by partial removal of the proximal enhancer. Overall, these data suggest that the 579 construct contains most of the regulatory elements responsible for SPACA6/miR-125a transcription.

**Figure 4:**
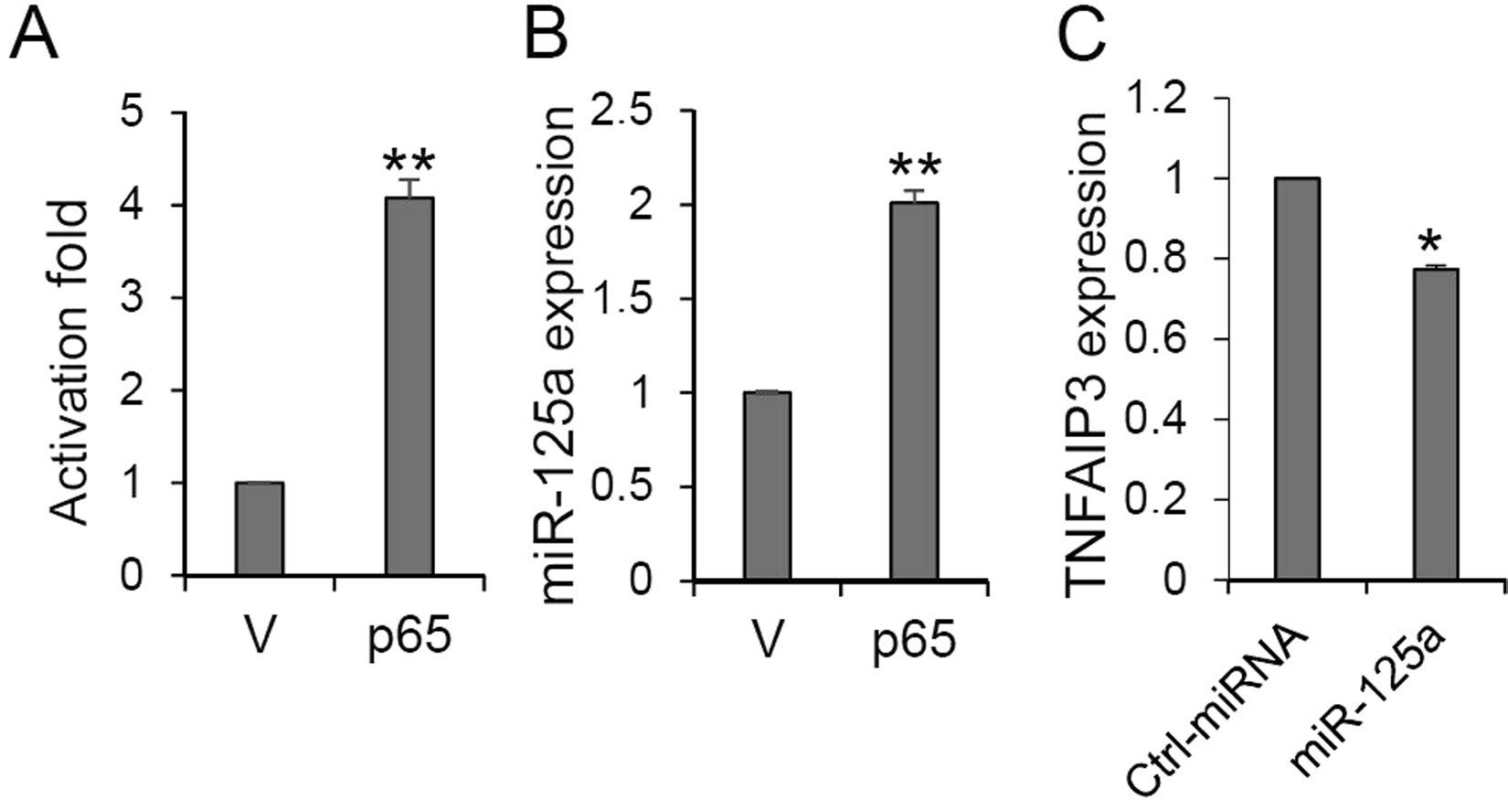
Effect of NF-kB on miR-125a expression. (**A**) Promoter activity of 579 construct was assayed in HepG2 cells following transfection of a p65 expressing plasmid (p65) or the parental vector (V). (**B**) miR-125a expression in HepG2 cells overexpressing p65. (**C**) TNFAIP3 expression in HepG2 cells transfected with miR-125a mimic or control mimic. Assays were performed in triplicate and expressed as mean ± SD; *p < 0.05 and **p < 0.01 at Student’s t-test.

Looking for positive regulators of miR-125a expression, we focused our attention on the 3′-end region of 579 segment, since its deletion reduced the promoter activity in both HepG2 and HuH-7 cells. In this region, de la Rica *et al*. recently reported the presence of a binding site for p65 subunit of NF-kB whose occupation stimulates miR-125a expression during osteoclast differentiation^46^. We then verified the effect of p65 on miR-125a expression in hepatic cells. Co-transfection of HepG2 cells with the 579 reporter construct along with a p65 expressing vector resulted in a 4-fold activation of luciferase activity compared to co-transfection with the parental vector (Fig. 4A). Later, the expression of miR-125a was evaluated, revealing a 2-fold upregulation by p65 (Fig. 4B). The lower extent of up-regulation of miR-125a compared to the induction of the isolated promoter may be due to post-transcriptional regulation by the RNA-binding protein Lin-28 that limits pre-miR-125a maturation^20^. Intriguingly, it has been reported that miR-125a constitutively activates the NF-kB pathway by targeting its negative regulator TNFAIP3 in diffuse large B-cell lymphoma^47^. We then verified this effect in HCC by transfection of a miR-125a mimic. This treatment significantly reduced the expression of TNFAIP3 (Fig. 4C), suggesting the occurrence of a positive self-regulatory loop whereby NF-kB p65 stimulates the transcription of miR-125a, that in turn downregulates TNFAIP3 with further activation of NF-kB pathway, thus strengthening miR-125a transcriptional activation. This effect may be functionally relevant since the pathogenesis of HCC has a common background in chronic inflammation and oxidative stress^48–50^; miR-125a induction by NF-kB may then limit the deleterious consequences of inflammation.

### Interplay between SPACA6 pre-mRNA splicing and miR-125a biogenesis

**Figure 5:**
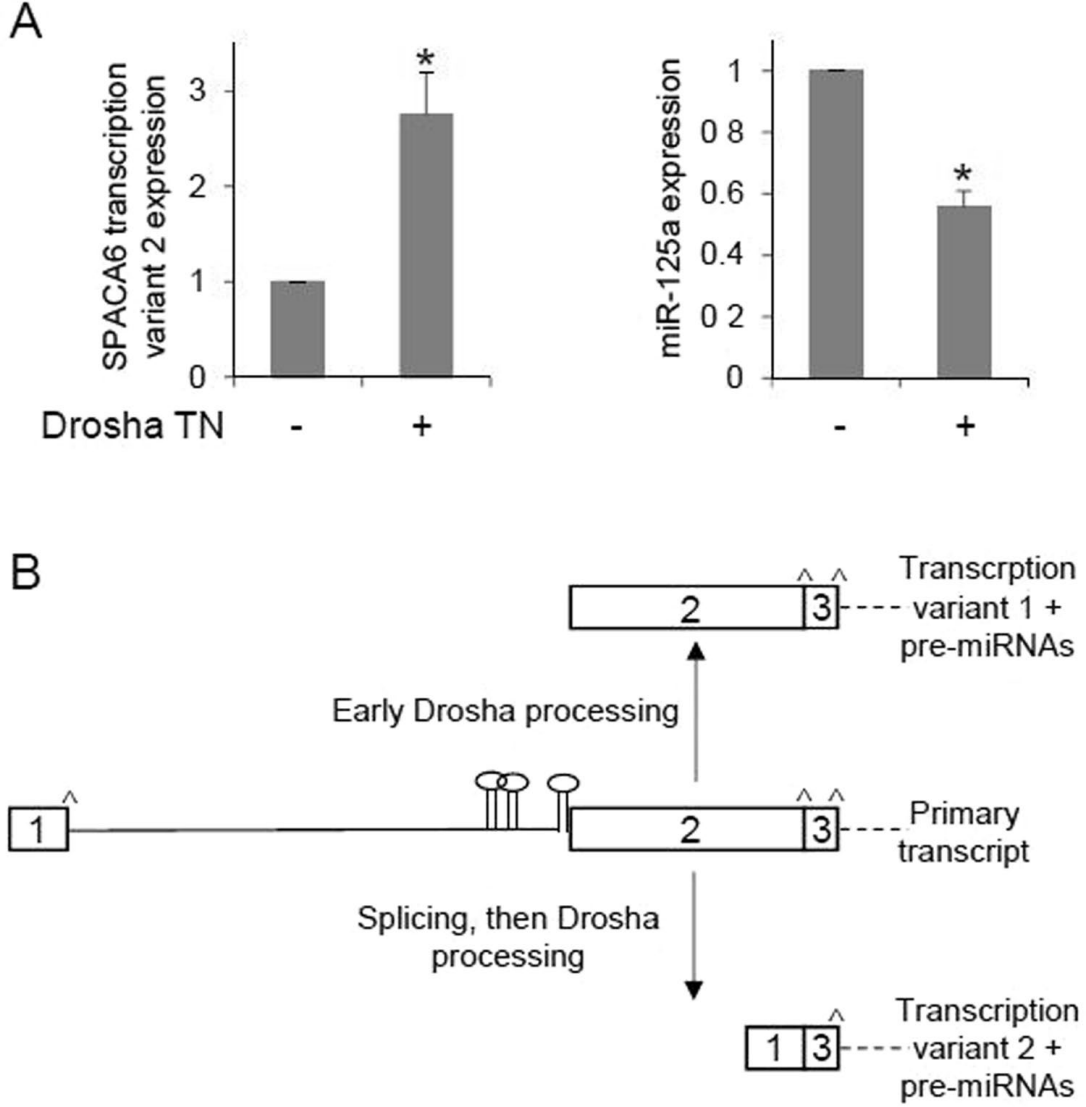
Role of Drosha in SPACA6 pre-mRNA processing. (**A**) HepG2 cells were transfected with a plasmid expressing trans-dominant-negative Drosha (Drosha TN) containing inactivating mutations in critical residues of the catalytic RNases domains; 48 h later, RNA from mock (−) and Drosha TN (+) transfected cells was extracted and expression of SPACA6 transcription variant 2 and miR-125a were evaluated by RT-qPCR. Assays were performed in triplicate and expressed as mean ± SD; *p < 0.05. (**B**) Model for maturation of SPACA6 primary transcript. Two pathways, depending on the timings of splicing and Drosha processing, lead to SPACA6 transcription variant 1 and 2. Only the 5′ region of the pre-mRNA is shown. ^Marks splice sites.

As shown in Fig. 1, pre-miR-125a hairpin is located within an intron of the newly assembled SPACA6 transcription variant 2, but its 3′-end is immediately adjacent to the 5′-end of a SPACA6 exon belonging to transcription variant 1. Variant 2 was recently identified with a deep RNA-sequencing approach after blocking the activity of Drosha, thus preventing pri-miRNA processing^45^. The authors of the work suggested that early processing by Drosha of SPACA6/miR-125a primary transcript would bypass the splicing of the first intron enhancing the accumulation of the transcription variant 1; otherwise, splicing of the first intron would yield transcription variant 2. We then attempted to validate these results in our experimental system and found that the transfection in HepG2 cells of a vector expressing trans-dominant-negative Drosha (Drosha TN)^51^ resulted in a three-fold enrichment of the SPACA6 variant 2 (Fig. 5A), indicating prevailing of the splicing on the primary transcript; on the other hand, the level of miR-125a was reduced, as expected as a consequence of the inhibition of the microprocessor activity (Fig. 5A).

Taken together, the data suggest the following miRNA expression pathway (Fig. 5B): miR-99b/let-7e/miR-125a are co-transcribed with SPACA6 from the promoter experimentally validated in this work; then, prevailing of splicing produces the SPACA6 transcription variant 2 and represents the first step of miRNA biogenesis and production of SPACA6 protein isoform 2, whereas prevailing of Drosha processing releases the SPACA6 transcription variant 1, leading to production of protein isoform 1. This way, timing of Drosha processing, prior or after splicing, affects pre-mRNA maturation leading to different transcripts and gene products. The biological significance of this process remains to be explained.

### Expression profiles of miR-125a

**Figure 6:**
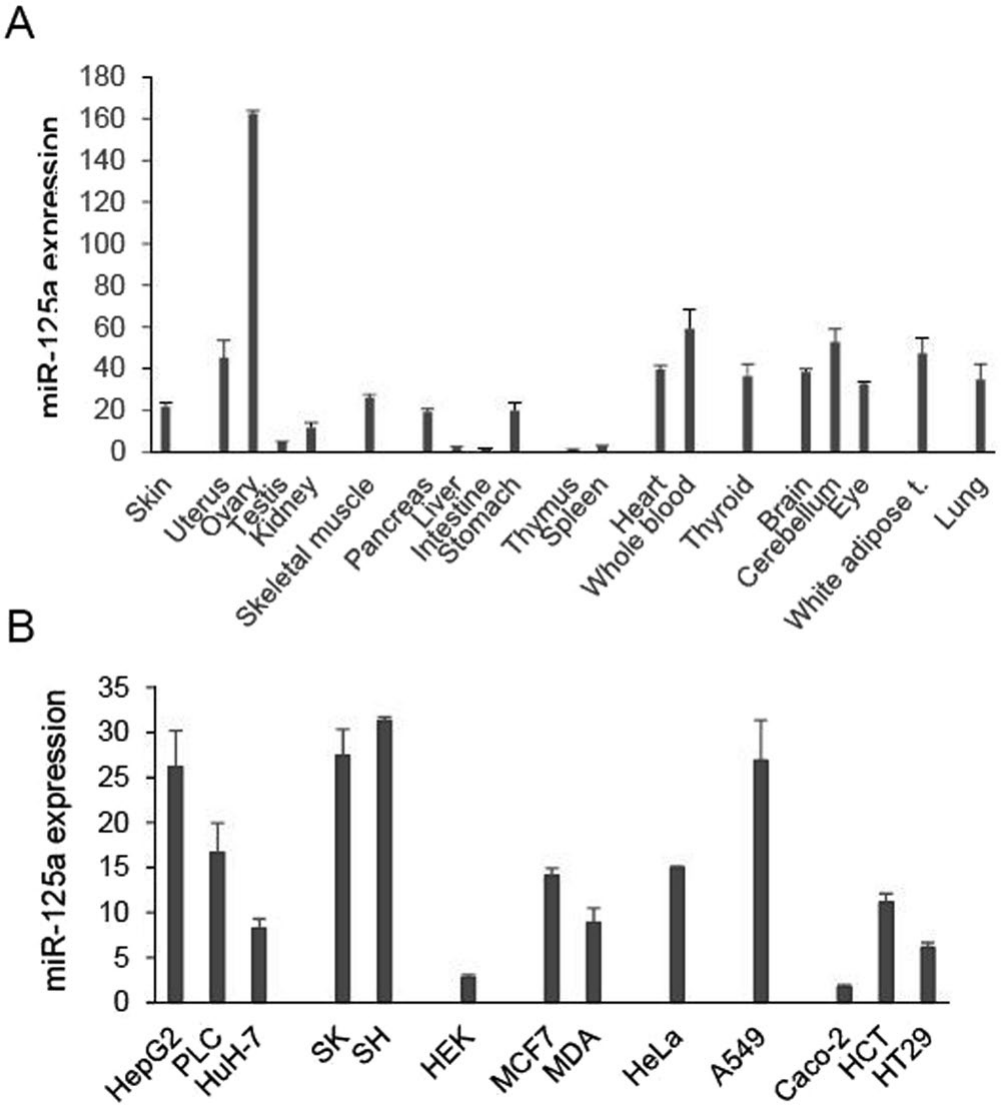
miR-125a expression in tissues and cell lines. (**A**) miR-125a expression was determined by qPCR in skin, uro-genital tract (uterus, ovary, testicles and kidney), skeletal muscle, gastrointestinal tract (pancreas, liver, intestine, stomach), lymphoid bodies (spleen, thyme), heart and whole blood, thyroid, eyes, cerebellum and brain, white adipose tissue and lung. (**B**) miR-125a expression was determined by qPCR in hepatocarcinoma cell lines HepG2, HuH-7, PLC/PRF/5 (PLC), neuroblastoma cell lines SK-N-BE(2)-C (SK) and SH-SY5Y (SH), HEK293 (HEK), breast cancer cell lines MCF7 and MDA-MB-453, HeLa cells, lung cancer A-549, colorectal adenocarcinoma Caco-2, HCT 116, HT-29. Assays were performed in triplicate and expressed as mean ± SD.

MiR-125a expression was analyzed in several murine tissues and resulted to be detectable in all samples, but with some variations. The highest expression was found in the ovary, but the miRNA was also well expressed in uterus, nervous system, heart, white adipose tissue, lung and thyroid (Fig. 6A). A lower level of expression was detected in the other tissues, such as the gastro-intestinal tract, skeletal muscle and skin. Profiling was then extended to cultured human cell lines, revealing an high expression of miR-125a in HepG2, neuroblastoma and lung cancer cells (Fig. 6B).

## Conclusions

Lin-4/miR-125a plays a fundamental role during development in controlling the expression of Lin-28 protein thus promoting phase transitions and cell differentiation in Nematodes, Insects and Mammals^17–20^. In this study we showed that miR-125a is widely expressed in the adult tissues, were we speculate it may modulate cellular sensitivity to mitogenic signals. With regard to the biogenesis of miR-125a, the collected data indicate that it belongs to an intronic cluster sharing with SPACA6 gene a TATA-less promoter provided with INR and DPE. Its activation by NF-kB, and the ability of miR-125a to downregulate TNFAIP3, provide a link between inflammatory response and miR-125a expression. This is consistent with the previous finding that exposure of macrophages to the fungal pathogen *Candida albicans* or bacterial lipopolysaccharides up-regulates miR-125a via NF-kB^52^. These results may also explain the increased expression of miR-125a observed in liver of patients with chronic hepatitis B^50^ and shed new light on the observed ability of miR-125a to counteract hepatitis B virus replication^48, 53–57^.

It is noteworthy that the isolated 579 bp genomic segment identified as SPACA6/miR-125a promoter is responsive to NF-kB in the luciferase reporter assay as the endogenous gene responds to NF-kB in driving miR-125a expression. This result validates the isolated promoter and make it suitable for the search of compounds able to increase its activity. This may be achieved by plate-based reporter assays employing secreted luciferases that enable real-time analysis of promoter activity in response to chemical compounds. Identified molecules may then provide a valuable tool in cancer research as promoter induction should also increase expression of the other members of the miRNA cluster, let-7e and miR-99b, both displaying an antiproliferative activity. Let-7e, in fact, belongs to the let-7 family of oncosuppressive miRNAs acting on key oncogenes, such as Ras, MYC and CDK6^58^ whereas miR-99b targets IGF-1R in human keratinocytes, mTOR in colorectal cancer cells, and FGFR3 in non-small lung cancer^59, 60^.

## Materials and Methods

### Ethics statements

Mice were maintained under specific pathogen-free conditions in the animal facility of the Department of Molecular Medicine and Medical Biotechnology. All animal experiments were performed in accordance with the regulations and guidelines of Italy and were approved by the ethical committee of the University of Naples Federico II. In accordance with institutional guidelines, mice were sacrificed using the CO_2_ method.

### Preparation of reporter plasmids for promoter testing

Genomic DNA was purified from HepG2 cells by the High Pure PCR Template Preparation kit (Roche). Genomic DNA segments were isolated by PCR using FastStart High Fidelity PCR System (Roche) with forward primers carrying a *Mlu*I restriction site and reverse primers carrying a *Sca*I restriction sites. PCR products were gel purified by Qiaquick® gel extraction kit (Qiagen) and cloned into *Mlu*I and *Sma*I restriction sites of pGL3-basic vector (Promega) to obtain the constructs shown in Figs 1 and 3. Primers were: 869 construct, 5′-CGACGCGTGAGACACAGATAATGCCCCACAG-3′ and 5′-AAAAGTACTACCCTCCCTTTCCGCAGAC-3′; 707 construct, 5′-CGACGCGT GTGAGTCTGCGGAAAGGGAG-3′ and 5′-AAAAGTACTAGTGTGGATTCCCTGGTCTGAG-3′; 1918 construct, 5′-CGACGCGTAATCCACACTCCAGCCCCTAAC-3′ and 5′-AAAAGTACTACACCTGCTTCCTACCTACCCTC-3′; 1219 construct, 5′-CGACGCGTGAGGGAGAGGAAGTGAGGAAAGAC-3′ and 5′-AAAAGTACTCAAGGAACCCAGGAGTCCAG-3′; 579 construct, 5′-CGACGCGT AGGACGGGGCGGAGAGG-3′ and 5′-AAAAGTACTACCCTCCCTTTCCGCAGAC-3′; 398 construct, 5′-CGACGCGTAGGACGGGGCGGAGAGG-3′ and 5′-AAAAGTACTCTTCTCCCCGACTCCCTCC-3′; *Mlu*I and *Sca*I sites are underlined. The 220 construct was obtained by digestion of the 384 construct with *Sma*I (restriction site within the genomic fragment) and *Hind*III (restriction site in the polylinker) and cloning the obtained fragment in the same sites of pGL3-basic vector. The 869mut construct with the mutated translation start codons described in Fig. 2 was obtained by site-directed mutagenesis with the PCR-based overlap extension method^61^ as described^62^. All the reporter constructs were sequenced to confirm their identity.

### Cell cultures

HepG2, HCT 116, HT-29, and SK-N-BE(2)-C cells were cultured in RPMI 1640 containing 10% fetal bovine serum, 2 mM L-glutamine, 50 U/ml penicillin and 100 µg/ml streptomycin; A-549, HeLa, HEK293, HuH-7, MCF-7, MDA-MB-453 and SH-SY5Y cells were cultured in DMEM containing 10% fetal bovine serum, 2 mM L-glutamine and the same antibiotics; Caco-2 and PLC/PRF/5 cells were cultured as HuH-7 except that medium contained 0.1 mM non-essential amino acid. Other procedures were performed as described^63^.

### Transfections and luciferase assays

The day before transfection, HepG2 cells were trypsinized and seeded in medium without antibiotics in 12-well plates. Transfections were then performed with cells at 80–90% of confluence by using 3 µl of Lipofectamine2000 (Invitrogen) for 1 µg of nucleic acids. For promoter testing, 1 µg of reporter plasmid or parental pGL3-basic vector were transfected along with 0.05 µg of phRL-TK (Promega), used to normalize transfection efficiency. The overexpression experiments were performed with 1.5 µg of plasmid expressing Drosha TN, a transdominant Drosha mutant^51^, or 1.5 µg of plasmid hRelA-HA-pBent2 expressing p65^64^. After 6 h, transfection mix was replaced with complete medium, and luciferase activity was recorded 48 h after transfection using the Dual-Luciferase Reporter Assay System (Promega). The firefly luciferase activity of the reporter plasmid was then normalized for transfection efficiency with the Renilla luciferase activity of the co-transfected phRL-tk plasmid, and the activity of the parental vector pGL3-basic was set to 1.

### RNA purification and real-time PCR analyses

Adult mouse tissues were freshly collected from one male and one female 8-week-old C57BL/6 J mice. Tissues were immediately homogenized using IKA T10 basic ULTRA-TURRAX homogenizer in an appropriate volume of QIAzol lysis buffer (Qiagen). Total RNA was extracted from cell cultures and from murine tissues by miRNeasy mini kit (Qiagen). MicroRNA-125a was quantified along with RNU6B (reference transcript) by RT-qPCR with TaqMan® miRNA assays from Applied Biosystems according to the manufacturer’s protocol. For quantification of SPACA6 and TNFAIP3 transcripts, total RNA was retrotranscribed by Transcriptor High Fidelity cDNA Synthesis Sample kit (Roche) using random examer primers. Then standard SYBR Green Real-time qPCR assays were performed with the following primers: SPACA6 transcription variant 2, 5′-GGGGAGAGGATGGAGAGC-3′ and 5′-TCATTTTCTCCGCAGCATC-3′^45^; TNFAIP3, 5′-TCAACTGGTGTCGAGAAGTCC-3′ and 5′-CCAAGTCTGTGTCCTGAACG-3′^46^; GAPDH (reference gene) 5′-GAAGGTGAAGGTCGGAGTC-3′ and 5′-GAAGATGGTGATGGGATTT-3′. Finally, the expression levels of miR-125a, SPACA6 and TNFAIP3 were normalized to their respective reference genes by using the 2^−ΔCt^ method and reported as arbitrary units (AU). Comparison of data sets was performed by Student’s t-test and a value of p < 0.05 was considered significant.
